# Robust Spin-Gapless Behavior in the Newly Discovered Half Heusler Compound MnPK

**DOI:** 10.3390/ma12193117

**Published:** 2019-09-25

**Authors:** Jiaxue You, Jieting Cao, Rabah Khenata, Xiaotian Wang, Xunan Shen, Tie Yang

**Affiliations:** 1 School of Physical Science and Technology, Southwest University, Chongqing 400715, China; youjx959@163.com (J.Y.); 13308348369@163.com (J.C.); xiaotianwang@swu.edu.cn (X.W.); 2 Laboratoire de Physique Quantique de la Matière et de Modélisation Mathématique, Université de Mascara, Mascara 29000, Algeria; khenata_rabah@yahoo.fr; 3 Science and Technology on Power Sources Laboratory, Tianjin Institute of Power Sources, Tianjin 300130, China

**Keywords:** first principles calculation, half Heusler compound, spin gapless

## Abstract

Spin gapless semiconductors have aroused high research interest since their discovery and a lot of effort has been exerted on their exploration, in terms of both theoretical calculation and experimental verification. Among different spin gapless materials, Heusler compounds stand out thanks to their high Curie temperature and highly diverse compositions. Especially, both theoretical and experimental studies have reported the presence of spin gapless properties in this kind of material. Recently, a new class of d^0^ − d Dirac half Heusler compound was introduced by Davatolhagh et al. and Dirac, and spin gapless semiconductivity has been successfully predicted in MnPK. To further expand the research in this direction, we conducted a systematical investigation on the spin gapless behavior of MnPK with both generalized gradient approximation (GGA) and GGA + Hubbard U methods under both uniform and tetragonal strain conditions by first principles calculation. Results show the spin gapless behavior in this material as revealed previously. Different Hubbard U values have been considered and they mainly affect the band structure in the spin-down channel while the spin gapless feature in the spin-up direction is maintained. The obtained lattice constant is very well consistent with a previous study. More importantly, it is found that the spin gapless property of MnPK shows good resistance for both uniform and tetragonal strains, and this robustness is very rare in the reported studies and can be extremely interesting and practical for the final end application. This study elaborates the electronic and magnetic properties of the half Heusler compound MnPK under uniform and tetragonal strain conditions, and the obtained results can give a very valuable reference for related research works, or even further motivate the experimental synthesis of the relative material.

## 1. Introduction

Since the discovery of spin gapless semiconductors, they have become of high interest in both scientific research and industrial application due to their very special electronic band structures [1,2,3,4]: In one spin direction, a small band gap is present similar to a semiconductor; in the other spin direction, the conduction band minimum exactly overlaps with the valence band maximum at the Fermi energy level, resulting in a zero band gap or gapless contact. Due to these particular band structures, spin gapless semiconductors have several remarkable properties [5,6,7]: No energy is required for electron excitation from the valence band to the conduction band; very high electron mobility; 100% spin polarization of both electrons and holes. Spin gapless behavior has been found in many materials, including both 2D and 3D materials [7]. These materials have very promising applications in the field of magnetoelectronics and spintronics [8,9,10,11]. 

Among different materials, Heusler compounds have attracted a lot of attention thanks to their high Curie temperature and extremely diverse compositions and, more importantly, both theoretical prediction and experimental confirmation have been reported for the presence of spin gapless properties in Heusler materials [4,5,6,7,8,12,13,14,15,16,17,18,19,20]. For example, Galanakis et al. [21] systematically investigated a series of inverse Heusler compounds from theoretical calculations and successfully identified several spin gapless full Heusler compounds, Ti_2_MnAl, Ti_2_CoSi, Mn_2_CoAl, Cr_2_ZnSi and so on. Further, Fecher et al. [22] realized Mn_2_CoAl polycrystalline by the arc melting method and demonstrated its spin gapless magnetic semiconducting behavior via experimental verification. Wang et al. [15] and Patel et al. [23,24,25] found that several Zirconium based full Heusler compounds have spin gapless band structures; besides, many quaternary Heusler compounds are spin-gapless [26,27], including CoFeMnSi, CoFeCrAl, CoMnCrSi, CoFeVSi and FeMnCrSb, in which CoFeMnSi has been experimentally synthesized [6,28,29]. Zhang et al. screened and identified 70 so far unreported spin gapless quaternary Heusler compounds from 12,000 possible candidates by high throughput technique [30]. A detailed review for the recent advances in the development of Heusler based spin gapless materials can be found in [7]. More and more research attention and effort are still continuing in this field.

Recently, a new class of d^0^ − d Dirac half Heusler compound has been proposed by Davatolhagh et al. [31] and it is defined by its compositions: d refers the 3D transition metal element and d^0^ stands for the metal element defined by the valence electronic configuration ns^1,2^, (n−1) d^0^. As a prototype, MnPK exhibits Dirac spin gapless semiconductivity. To further expand the research in this direction, we conducted a systematical investigation on the spin gapless behavior of d^0^ − d half Heusler compound MnPK with both GGA and GGA + U methods by first principle calculations. The spin gapless property is confirmed even under different Hubbard U values. In particular, the effects of uniform and tetragonal strain conditions is examined, which are very important prospects for the real application situation. Results clearly show the robustness of the spin gapless property in this material against both strains. This study could give a very valuable reference for related research works or even further motivate the experimental synthesis of the relative material.

## 2. Computational Methodology

The electronic and magnetic properties of half Heusler compound MnPK have been studied with the first principles calculations based on density functional theory [32]. The Cambridge Serial Total Energy Package (CASTEP) code [33] has been applied with the pseudo potential plane wave methods. The Perdew-Burke-Ernzerhof (PBE) functional within the generalized gradient approximation (GGA) [34] and the ultrasoft pseudo potential [35] are selected for the exchange-correlation potential and the interaction between the atomic core and the valence electrons, respectively. The configurations for valence electron of P(3s^2^3p^3^), K(4s^1^) and Mn(3d^5^4s^2^) were selected, respectively. After initial convergence test, the plane-wave cutoff energy is set as 500 eV and a k sampling mesh of 12 × 12 × 12 Monkhorst-Pack grid is selected in the Brillouin zone. The energy converge tolerance for the self-consistent field calculation is set within 1 × 10^−6^ eV/atom.

## 3. Results and Discussion

### 3.1. Crystal Structure and Equilibrium Lattice

**Figure 1:**
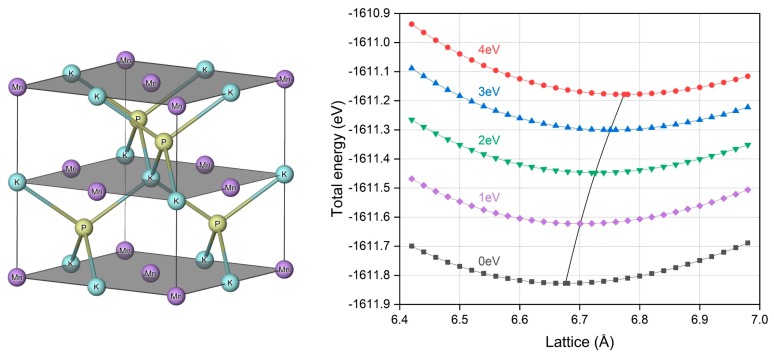
The crystal structure of half Heusler compound MnPK and the calculated total energy under different lattice constants with different Hubbard U values.

In general, for the half Heusler compound, it has the generic formula of XYZ [36,37,38,39,40], where X and Y are two different transition metal elements and Z is a main group element. Its crystal structure adopts the non-centrosymmetric C1_b_ arrangement ($F\bar{4}3M$, No. 216) with three interpenetrating face centered cubic sublattices defined by three Wyckoff coordinates: A (0,0,0), B (0.25,0.25,0.25) and C (0.5,0.5,0.5). This structure is very similar to the zinc blende one, except the B site is void. For the currently study d^0^ − d half Heusler MnPK, the A, B and C positions are occupied by Mn, P and K atoms, respectively, and its corresponding crystal structure in a one-unit cell is illustrated in Figure 1. In order to obtain the crystal structure at ground state and the corresponding equilibrium lattice constant, the total energy per unit cell is computed under variable lattice and the result is reported in Figure 1. Note that the Mn atom is from the transition metal elements and the GGA + U method is employed to better describe the strong correlation effect for its d electrons. The different onsite Coulomb energies are applied for the U values, as indicated in Figure 1, and 0 eV stands for the case without U applied. It is seen that the total energy is increased with Hubbard parameter U and it is obviously caused by the introduced Coulomb interaction. 

By applying polynomial curve fitting of the total energies and lattice constants, we can determine the equilibrium lattice constants with the total energy minimization and the lattice values under different U values are summarized in Table 1. The variation of lattice constants with different U values is displayed in Figure 1, and it is seen that the lattice increases with larger U values, which is from the stronger Coulomb repulsion. The derived lattice constants are in a very good agreement with previous theoretical results [31].

### 3.2. Electronic and Magnetic Properties

**Figure 2:**
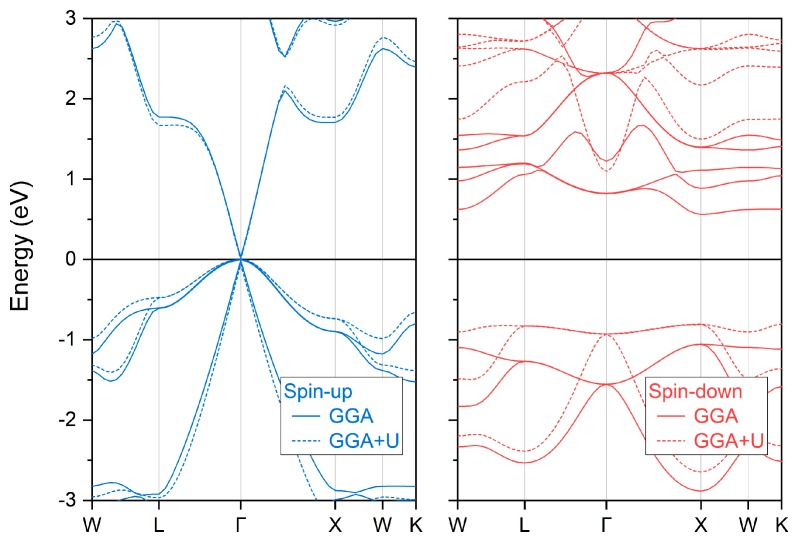
The calculated spin-polarized electronic band structure of half Heusler compound MnPK at the corresponding equilibrium lattice constant. For GGA + U method, 4 eV onside Coulomb energy is applied.

**Figure 3:**
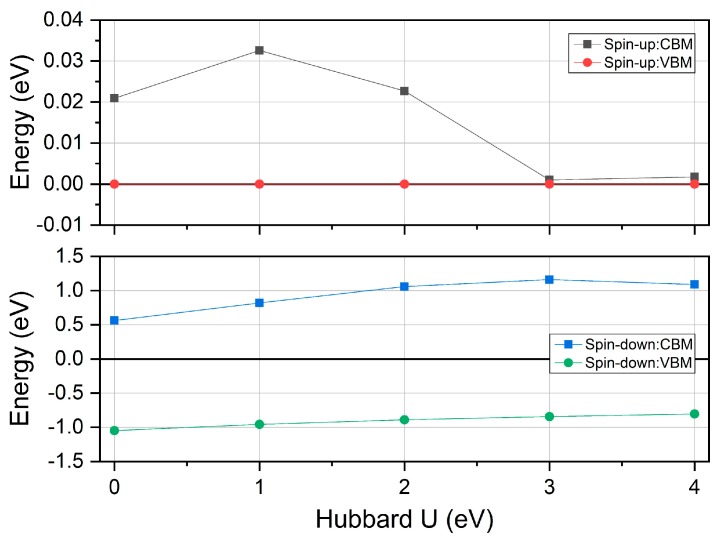
The calculated conduction band minimum (CBM) and valence band maximum (VBM) in both spin directions under different Hubbard U values for the half Heusler compound MnPK.

With the obtained equilibrium lattice constants of half Heusler compound MnPK, we can calculate its electronic and magnetic properties. Figure 2 displays the electronic band structure in both spin directions. We can clearly observe that there exists an energy band gap in the spin-down channel whereas the conduction band minimum and valence band maximum directly touch each other at the Γ point of the Fermi energy level in the spin-up channel. This band structure exactly features the spin gapless semiconducting behavior. Note that the band structures of both GGA and GGA + U methods have been calculated at their corresponding lattice constants. It is seen that there are some variations of the band structure in both spin directions with Hubbard U applied—in particular, the band structure in the spin-down direction is shift upward considerably. Specifically, for the band structure around the Fermi energy value, we calculate the variation of the conduction band minimum (CBM) and the valence band maximum (VBM) with different U values and the results are shown in Figure 3. Note the different scales in the vertical axis for the two spin directions. It is found that the CBM and VBM in the spin-down direction have been pushed upwards with U value increase; however, the band gap is still preserved. For the spin-up direction, the VBM stays constantly at the Fermi level but there is small variation for the CBM. Even with these changes, the spin gapless feature is maintained under different Hubbard U values for the half Heusler compound MnPK. The reason has been analyzed previously [31] that the VBM and CBM in the spin-up direction is mainly contributed by the P 3p, P 3s, and Mn 4s states, and thus the inclusion of Hubbard U has no effect on the electronic band near the Fermi level.

**Figure 4:**
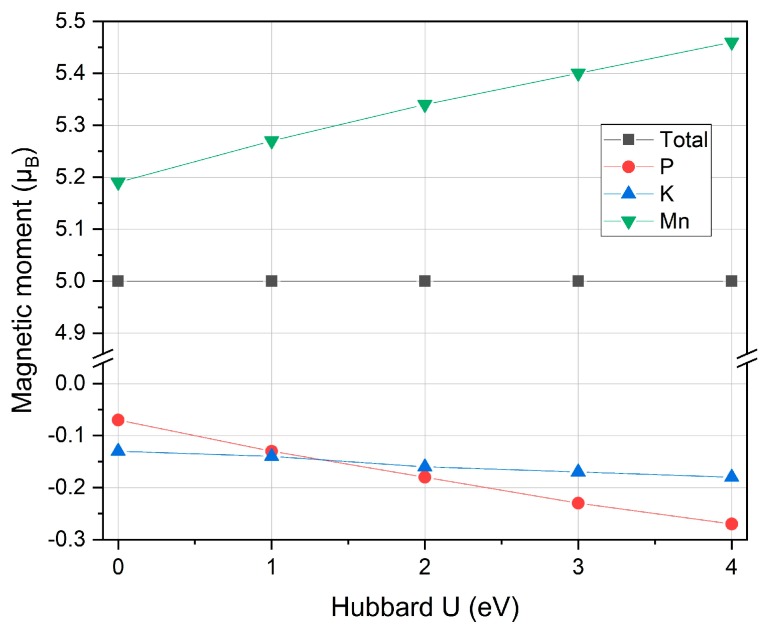
The calculated total and partial magnetic moments under different Hubbard U values for the half Heusler compound MnPK.

The spin gapless band structure of MnPK has several remarkable properties: multiple degenerate gapless points are present; there are both linear and parabolic gapless band shapes; the gapless points are all direct-band contacting. Especially for the linear structure, it is Dirac spin gapless with massless carrier transportation, which is very interesting for spintronic application [41]. At the obtained lattice constants, we further calculated the total and partial magnetic moments under different U parameters and the results are reported in Table 1 and plotted in Figure 4. The total magnetic moment of 5.00 μ_B_ is mainly contributed by the Mn atom and its integral value follows the Slater-Pauling rule [42] in the form of M_T_ = Z_T_ − 8, where the M_T_ is the total magnetic moment per formula unit, and Z_T_ is the total number of valence electrons per formula unit, which is 13 for MnPK. The magnetic moments of P and K are very small and antiparallelly aligned with the Mn atom. The total magnetic moment does not change with different U values whereas the partial atom-resolved magnetic moments have varied, which is probably caused by the increased lattice constants under larger Hubbard U values.

### 3.3. Uniform Strain

**Figure 5:**
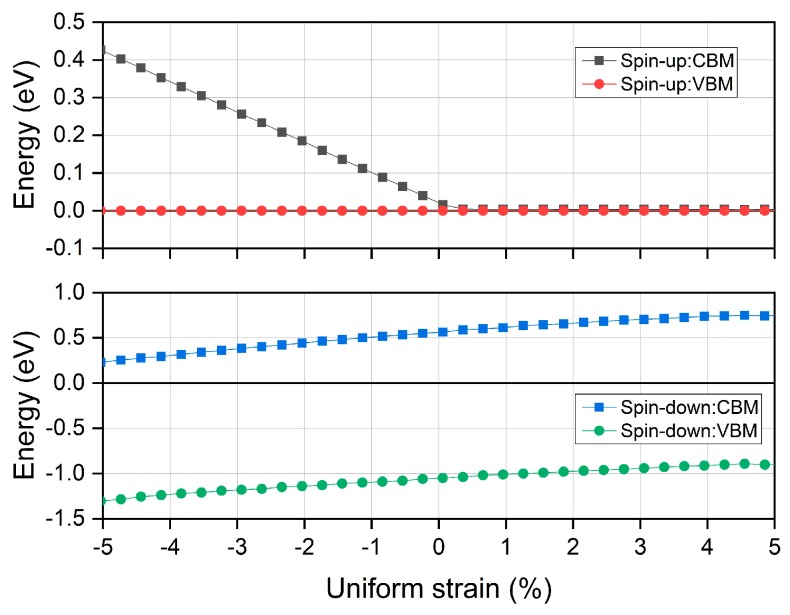
The calculated total and partial magnetic moments under different uniform strains for the half Heusler compound MnPK.

For Heusler compounds, their physical properties are closely correlated with the material structure and any possible distortions could lead to significant changes. In order to evaluate the stability of the spin gapless feature in the half Heusler compound MnPK, we further examined the electronic band structure under uniform strain condition by simply varying the lattice from −5% to 5% with respect to the equilibrium constant. Although the half Heusler compound MnPK has not been experimentally synthesized, the spin gapless property of this newly discovered d^0^ − d half Heusler is still worth investigating. It should be mentioned that there is only the GGA method without the onsite Coulomb correction applied for the following analysis. Since the spin gapless behavior is focused on, we only consider the variation of the conduction band minimum and the valence band maximum under different uniform strains. The calculated results are shown in Figure 5.

It is found that the CBM and VBM in the spin-down channel continuously increase with the uniform strain from −5% to 5% yet there is no crossing with the Fermi energy level, meaning the band gap is preserved at currently applied strain range. For the spin-up channel, there is only variation of the CBM at the negative strain side, a small band gap is opened up with lattice contraction, and the VBM maintains unchanged at the Fermi level. Because there is only a linear band dispersion part around the Fermi energy level in the spin-up direction as shown in Figure 2, this variation of CBM in the spin-up direction is from the linear band shape, and thus it shows that the Dirac degeneracy point is not related with the crystal symmetry as revealed in other materials [43]. In combination of both spin directions, the spin gapless feature is kept under positive uniform strain side. 

**Figure 6:**
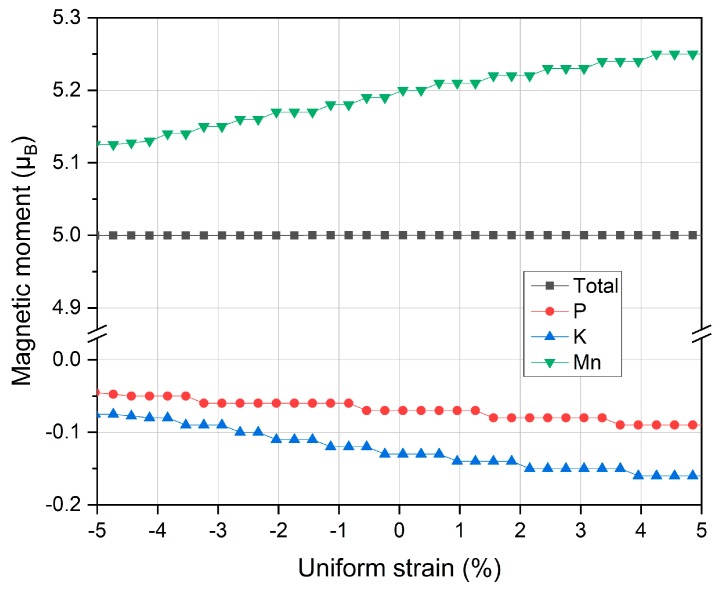
The calculated total and partial magnetic moments under different uniform strains for the half Heusler compound MnPK.

The magnetic property under uniform strain has also been studied and the calculated total and partial magnetic moments are plotted in Figure 6. Note the different scales of the two parts in the vertical axis. Throughout the whole uniform strain range, the total magnetic moment remains constant of 5.00 μ_B_ yet partial magnetic moments all increase (absolute value) from lattice contraction at the negative uniform side to the lattice expansion at the positive uniform side. In comparison, Mn atom has much larger moment variation than P and K atoms. With lattice decrease, the valence electrons become more delocalized and thus the atomic magnetic moments diminish, and vise verse.

### 3.4. Tetragonal Strains

**Figure 7:**
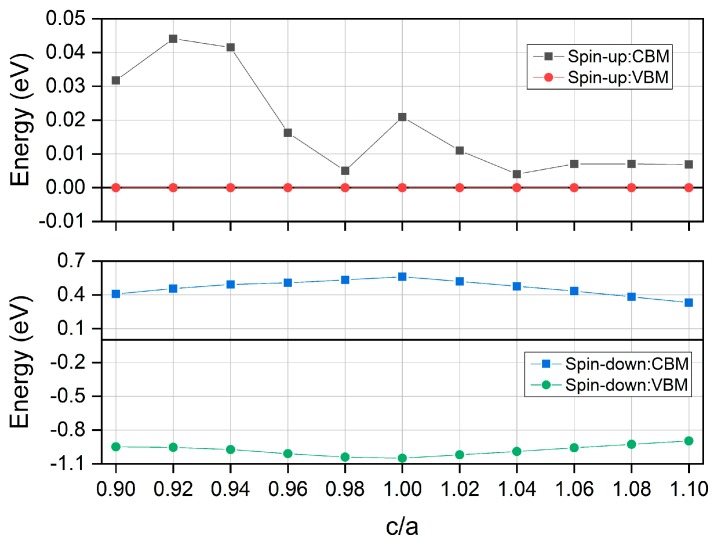
The calculated conduction band minimum (CBM) and valence band maximum (VBM) for the half Heusler compound MnPK in both spin directions under different tetragonal strains.

In this section, we further investigate the effect of tetragonal strains on the electronic and magnetic properties of the half Heusler compound MnPK. It should be pointed out that the tetragonal distortion is considered as the c/a ratio and the unit cell volume is preserved constantly at the equilibrium state. When c/a is equal to 1, there is no distortion and the crystal structure is cubic; when c/a is different from 1, the tetragonal distortion is applied. The tetragonal strain is considered as the c/a ratio from 0.90 to 1.10. The calculated CBM and VBM in both directions under tetragonal strain distortion are shown in Figure 7. Note the different scales in the vertical axis for the two spin directions. For the spin-down channel, the CBM is always decreased and the VBM is always increased when the c/a ratio is varied from 1. Thus, the band gap in the spin-down channel is decreased when the cubic crystal structure undergoes tetragonal distortion. For the spin-up direction, there is a slight variation only for the CBM and the VBM stays at the Fermi energy level. Considering the very small energy gap in the spin-up direction, the spin gapless behavior is maintained for the whole tetragonal distortion range.

**Figure 8:**
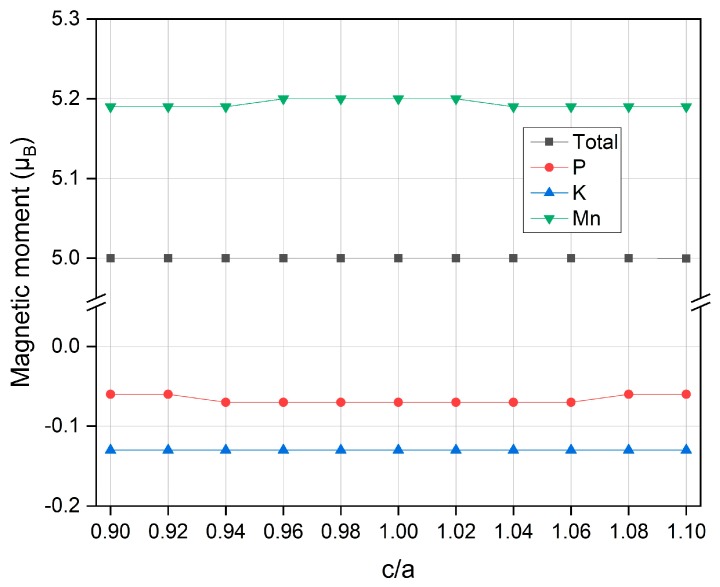
The calculated total and partial magnetic moments for the half Heusler compound MnPK under different tetragonal strain.

This robust spin gapless behavior against both strains may be related with the lack of spatial inversion symmetry found in half Heusler as proposed by de Groot et al. [44]. However, more research investigations should be needed to clarify this point. The total and partial magnetic moments under tetragonal strain are also calculated and the results are displayed in Figure 8. It is clearly shown that the total magnetic moment remains unchanged even there are some small variations for the partial moments. When the c/a ratio is different from 1, there is very small moment decrease for all atoms (absolute values) and this indicates that tetragonal distortion reduces atomic magnetic moments.

## 4. Conclusions

In this work, the electronic and magnetic properties of the half Heusler compound MnPK have been systematically investigated with the first principles calculation based on density functional theory. To better describe the 3D transition metal element Mn, both GGA and GGA + U methods have been applied and results all show the spin gapless behavior in this material as revealed previously. Different Hubbard U values have been considered and they mainly affect the band structure in the spin-down channel while the spin gapless feature in the spin-up direction is maintained. The obtained lattice constant is very well consistent with a previous study. Furthermore, the effects of uniform strain and tetragonal strain on the electronic and magnetic properties have been examined. It is found that the spin gapless property of MnPK remains for the lattice expansion at the positive uniform strain side and also throughout the whole tetragonal distortion at currently applied range. The total magnetic moment keeps constant of 5 μ_B_ with both applied uniform and tetragonal strains. The robustness of the spin gapless property against both strains found in this material is very rare and can be extremely interesting and useful for the final end application. This study elaborates the electronic and magnetic properties of the half Heusler compound MnPK under uniform and tetragonal strain conditions, and obtained results can give a very valuable reference for relative research works, or even further motivate the experimental synthesis of this material.

## Figures and Tables

**Table 1:** The calculated equilibrium lattice constants and the corresponding total and atom-resolved magnetic moments of half Heusler compound MnPK under different Hubbard U values.

Compound	U (eV)	Lattice (Å)	Magnetic Moment (μ_B_)			
			Total	P	K	M
MnPK	0	6.676	5.00	−0.07	−0.13	5.19
	1	6.701	5.00	−0.13	−0.14	5.27
	2	6.726	5.00	−0.18	−0.16	5.34
	3	6.751	5.00	−0.23	−0.17	5.40
	4	6.774	5.00	−0.27	−0.18	5.46
